# Origins of cancer: ain’t it just mature cells misbehaving?

**DOI:** 10.1038/s44318-024-00099-0

**Published:** 2024-05-21

**Authors:** Charles J Cho, Jeffrey W Brown, Jason C Mills

**Affiliations:** 1 Section of Gastroenterology and Hepatology, Department of Medicine, Baylor College of Medicine, Houston, TX USA; 2 Division of Gastroenterology, Department of Medicine, Washington University in St. Louis, School of Medicine, St. Louis, MO USA; 3 Department of Pathology & Immunology, Baylor College of Medicine, Houston, TX USA; 4 Department of Molecular and Cellular Biology, Baylor College of Medicine, Houston, TX USA

**Keywords:** Differentiation, Multicellularity, p53, Plasticity, Stem Cell, Cancer, Development, Evolution & Ecology

## Abstract

A pervasive view is that undifferentiated stem cells are alone responsible for generating all other cells and are the origins of cancer. However, emerging evidence demonstrates fully differentiated cells are plastic, can be coaxed to proliferate, and also play essential roles in tissue maintenance, regeneration, and tumorigenesis. Here, we review the mechanisms governing how differentiated cells become cancer cells. First, we examine the unique characteristics of differentiated cell division, focusing on why differentiated cells are more susceptible than stem cells to accumulating mutations. Next, we investigate why the evolution of multicellularity in animals likely required plastic differentiated cells that maintain the capacity to return to the cell cycle and required the tumor suppressor p53. Finally, we examine an example of an evolutionarily conserved program for the plasticity of differentiated cells, paligenosis, which helps explain the origins of cancers that arise in adults. Altogether, we highlight new perspectives for understanding the development of cancer and new strategies for preventing carcinogenic cellular transformations from occurring.

## Abstract (web summary)

This review summarises emerging views on how differentiated cells become cancer cells and contribute to malignancies due to accumulating mutations, aberrant tissue repair programs and hijacked cell state plasticity.

## Graphical abstract

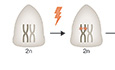

## Introduction

Since the mid-19th century, when Rudolf Virchow popularized the idea that *omnis cellula e cellula* (Virchow, 1860), it has been broadly accepted that any cell in an organism, including cancer cells, must have arisen from another cell. This paradigm naturally led to fundamental questions about the cellular origin of cancer: Which cells are susceptible to transforming into cancer? What causes the transformation, and how does it occur?

Here, we will give differentiated cells their due as cells of origin for cancer. We will discuss how the vast majority of cells in every organ are differentiated cells performing their normal physiological functions and that most organs (e.g., pancreas, kidney, and liver) do not have a dedicated stem cell population despite being susceptible to malignancy, whereas some organs that have active stem cells (e.g., small intestine) rarely undergo malignant transformation. We will discuss in depth why differentiated cells are more prone to accumulate mutations that can lead to transformation. Next, we will provide evidence on the plasticity and proliferative potential of differentiated cells that have existed throughout evolution. Indeed, the recruitment of differentiated cells as facultative stem cells may have evolved as a means to facilitate regeneration after injury. We will discuss the evolutionarily conserved features of a cellular program, paligenosis, that regulates how differentiated cells are recruited back to a progenitor state. Finally, we will explore how understanding plasticity and paligenosis in tumorigenesis might eventually help us to prevent tumors, improve the treatment of existing tumors, and understand the relationship between tumors and aging.

### The history of the cancer cell of origin

Prior to broad acceptance of Virchow’s *omnis cellula e cellula* (Virchow, 1860), numerous wide-ranging theories existed to explain the origins of cancer (see Box 1). By the early 1900s, these had largely coalesced around the three general cancer cell-of-origin candidates as articulated by pathologist George Adami: (1) tissue stem cells; (2) latent embryonic cells; (3) mature, differentiated cells (Adami, 1900). However, by the end of the 20th century, the professional tissue stem cell explanation of cancer-originating cells had achieved such dominance that the notion that differentiated cells could change their fate and become metaplastic, let alone tumors, was rarely considered (Bickenbach, 1981; Blanpain, Horsley et al, 2007; Morrison, Uchida et al, 1995; Potten and Loeffler, 1990). The hegemony of the stem cell origin theory appears to have derived from the homeostatic characteristics of stem cells, namely that they are proliferative and thus could accumulate and perpetuate mutations, eventually losing normal checks on proliferation and fueling clones that can expand indefinitely and thereby serve as the source of cancer.

**Figure 1:**
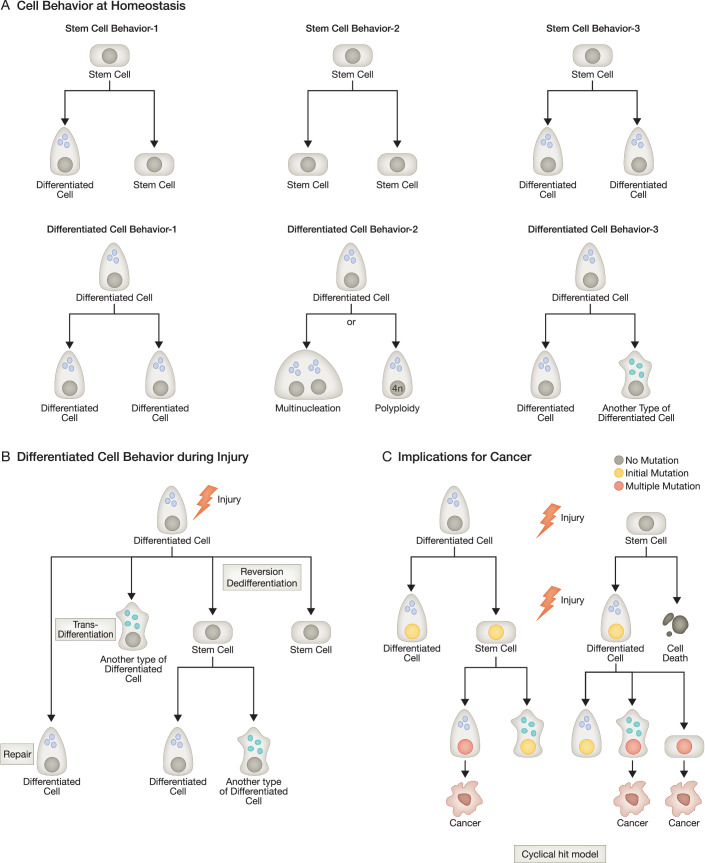
Various patterns of stem and differentiated cell behavior under different conditions. (**A**) Proposed cell behaviors in homeostasis. The canonical asymmetric stem cell self-renewal model (upper, left) has been augmented by recently proposed alternative behaviors, such as epithelial stem cells generating two stem cells or two differentiated cells (upper, middle, and right panels). Differentiated cells can (i) autoduplicate (lower, left), (ii) become multinucleated or polyploid (lower, middle), or (iii) give rise to another cell type via dedifferentiation or transdifferentiation (lower, right), although they are usually mitotically quiescent without injury. (**B**) Differentiated cell behavior during injury. After injury, differentiated cells can enter the cell cycle and become actively involved in replenishing the lost components in the given tissue, such as through paligenosis. (**C**) Implications for tumorigenesis. Through the cyclical hit model, differentiated cells go through cycles of differentiation and redifferentiation, acquiring mutations, increasing their potential to serve as a source of cancer. Note: “stem cell” in (**B**, **C**) refers to any cell with a proliferative, dedifferentiated, progenitor-like phenotype (i.e., not limited to true, professional, multipotent stem cells, which as discussed in the manuscript do not exist in most tissues).

However, in recent years, there has been a surge of ideas that have changed our perspective, as it has become abundantly clear that (1) most tissues don’t have a dedicated stem cell and (2) differentiated cells can proliferate, become plastic, and then serve as a new source of tissue regeneration and tumors. In the next section, we will compare the characteristics and relative abundance of adult stem cells and differentiated cells, which will point to the reasons for this resurgence (Fig. 1).

Box 1 History of cancer cell of origin Before the idea of all cells coming from existing cells had been broadly accepted as applying to cancer as well as phenotypically normal tissue at homeostasis, there were widely varying ideas about cancer’s origins. Some believed that cancer cells were not of human origin but instead bacterial or parasitic organisms akin to other infectious agents that form tissue (Warren, 1891), or cancer formation was facilitated by parasites invading and commandeering cells (Russell, 1890). Snow, in particular viewed cancer cells as amoeba-like parasites engulfing their neighbors (Snow, 1898). A series of studies by Adamkiewicz showed how injecting carcinoma cells into rabbit brains caused rapid spread of the cells throughout the body and death of the rabbit, such that the cancer cells acted like actual amoeba-like parasites, causing sepsis and releasing toxins (Adamkiewicz, 1893; Keating, 1913). Interestingly, Adamkiewicz went on to try to isolate carcinoma toxin (which he called “cancroin”) from the carcinoma organism (which he called Coccidium sarcolytus) in an attempt to use cancroin as a vaccine, à la Pasteur and Koch, to cure cancer (Pawlina and Maciejewska, 2002; Skalski and Zembala, 2005). Though his work had its proponents, it was largely discounted with Adamkiewicz himself eventually leaving academic medicine. By the onset of the 20th century, thinking in the field had coalesced around the theories summarized by Adami that adult cancers arise either from (1) stem cells, (2) residual embryonic tissue, or (3) differentiated cells.

Adami’s 2nd cancer origin theory is derived from Cohnheim’s hypothesis (Cohnheim, 1882) that cancers arise from embryonic rests of tissue that subsequently get reactivated to form tumors. Connheim’s work had its proponents and opponents for decades. In support, for example, Max Askanazy was able to produce teratomas in rats after implantation of embryonic cells (Askanazy, 1907). However, most cancer scholars by the 20th century had reasoned that evidence for this origin existed for only a small fraction (estimated at 2% by Snow) of adult tumors that had blast-like or germ-cell-like characteristics as in teratomas (Snow, 1898; Wilks and Moxon, 1889).

It was also beginning to be noted by the fin-de-siècle that cancers tend to be associated with or arise from precursor lesions comprising not-yet malignant cells that were from lineages not typically seen in the tissue and/or organized differently from that of the homeostatic pattern. Thus, the cells that eventually transform into cancer may first change phenotype and give rise to such lesions before they progress further toward malignancy. Virchow coined the term metaplasia for a transformal response resulting in a lesion characterized by the emergence of a population of non-dysplatic cells, typical for another tissue but ectopic to the tissue where the lesion is (Virchow, 1884). Within a few decades, the term metaplasia was largely—though not always—applied to lesions with an increased risk of progression to cancer, which distinguishes metaplastic lesions from benign rests which are embryonic islands of ectopic tissue. A century ago, Ménétrier and others reported that metaplastic precursor lesions typically arose in the setting of chronic inflammation, and it was believed that such metaplasias might represent a tissue adaptation to the insult (Ménétrier, 1926). Thus, cancer biologists reasoned that cancer cells arise from metaplastic cells that in turn derived from normal homeostatic differentiated cells subject to chronic damage. From that time on, the consequential obvious questions have not been resolved: which normal, nonmalignant cells launch themselves along this metaplasia to dysplasia route? Which cell types are the ones that undergo this plasticity that can then progress to cancer?

### The case for differentiated cells over adult tissue stem cells as cells of origin for cancer

#### Differentiated cells are capable of cell division, but in a unique way

First, despite tremendous enthusiasm and growth in understanding stem cells in the last few decades, it has become apparent that dedicated stem cells are uncommon in adult animals, and only a small fraction of tissues possess them. Stem cells are constitutively mitotically active cells that can both self-renew as well as differentiate into other daughter lineages (Fig. 1A, upper left). During homeostasis, they are largely confined to tissues that continuously shed cells, such as blood, skin, and luminal alimentary tracts in adult tissue (Barker, Huch et al, 2010; Barker, van Es et al, 2007; Blanpain and Fuchs, 2006; Ogawa, 1993). Indeed, most adult organs don’t have constitutively mitotic, undifferentiated cells; they have evolved in a more cost-effective way to maintain cell census and replenish cell loss during homeostasis and even after injury.

For example, the adult exocrine and endocrine pancreas have only a scant need for cell proliferation to preserve the census of long-lived cells. Whatever cells are lost at homeostasis are replaced infrequently by autoduplication (see Box 2 for term definitions) of existing differentiated acinar or islet cells without the need for a dedicated, undifferentiated stem cell (Fig. 1A, lower left) (Desai, Oliver-Krasinski et al, 2007; Dor, Brown et al, 2004; Teta, Rankin et al, 2007). Likewise, autoduplication of hepatocytes and cholangiocytes is mostly responsible for the homeostatic maintenance of liver parenchymal cells (Duncan, Dorrell et al, 2009; Malato, Naqvi et al, 2011; Ponder, 1996; Stanger, 2015; Yanger, Knigin et al, 2014). Chief cells and neuroendocrine cells in the corpus of the stomach, along with serotonin, gastrin, and somatostatin-producing cells in the antrum, largely autoduplicate to maintain their own census at homeostasis (Burclaff, Willet et al, 2020; Dayal and Wolfe, 1991; Kubben and Bosman, 1989; Lehy, 1982; Ryberg, Tielemans et al, 1990; Sundler, Ekblad et al, 1991; Tielemans, Håkanson et al, 1989; Tielemans, Willems et al, 1990). Salivary gland acinar cells, another exocrine secretory cell, also undergo occasional autoduplication during homeostasis to maintain their numbers (Aure, Konieczny et al, 2015; Denny, Chai et al, 1993). Mammalian adult kidney (e.g., cells in the proximal tubule) is maintained by occasional tubulogenesis of fate-restricted clones at homeostasis (Humphreys, Czerniak et al, 2011; Rinkevich, Montoro et al, 2014; Vogetseder, Palan et al, 2007). In the pituitary, corticotroph cells autoduplicate to maintain their cell population at homeostasis or after ablative destruction (Langlais, Couture et al, 2013).

In short, most organs do not have bona fide adult stem cells but are still capable of replacing cells at homeostasis. Even in tissues like the epithelial lining of the gastrointestinal tract, which does harbor constitutive adult stem cells, those stem cells are not necessarily responsible for actively regenerating all the epithelial cells in the organ. For example, as mentioned above, in the body of the stomach, the exocrine chief cells at the base of the gastric glands appear to maintain their own numbers by autoduplication at homeostasis, undisturbed by adult stem cell populations. After injury, the mature differentiated chief cells reprogram to progenitor-like cells to replace their own lost numbers (Burclaff et al, 2020; Han, Fink et al, 2019).

#### Differentiated cells have a long half-life and low cell turnover rate

Second, Because the majority of differentiated cells have a long half-life and a low cell turnover rate, they are more prone to developing, and thereafter accumulating, chromosomal abnormalities through mutation-prone repair mechanisms. In mice, differentiated pancreatic acinar cells are quiescent at homeostasis and can live for months (Magami, Azuma et al, 2002b, Sangiorgi and Capecchi, 2009); similarly, fully differentiated stomach corpus chief cells live many months (Burclaff et al, 2020). Likewise, hepatocyte turnover in adult liver ranges from 200 to 400 days (Duncan et al, 2009; MacDONALD, 1961; Magami, Azuma et al, 2002a, Stanger, 2015), while Paneth cells in the intestine have a half-life of 57 days (Ireland, Houghton et al, 2005). The average half-life of ciliated airway epithelial cells in the trachea and lung can be as long as six and seventeen months, respectively (Rawlins and Hogan, 2008). In line with this, alveolar epithelium is terminally differentiated and mitotically quiescent at homeostasis; type I alveolar cells have an approximate lifespan of 120 days (Herzog, Brody et al, 2008; Kotton and Morrisey, 2014). Finally, the turnover rate of adult prostate epithelium is extremely slow (Choi, Zhang et al, 2012), as is the kidney which comprises mostly post-mitotic,quiescent cells (Humphreys, Valerius et al, 2008; Messier and Leblond, 1960; Thomasova and Anders, 2014).

On the other hand, the small intestine villus completely regenerates every 4–5 days (Barker et al, 2007; van der Flier and Clevers, 2009). Hematopoietic stem cells (HSCs) show a high turnover rate; about 6–8% of self-renewing HSCs enter the cell cycle per day, and 99% of HSCs divide on average every 57 days (Cheshier, Morrison et al, 1999; Kiel, He et al, 2007). In the stomach, actively proliferating progeny of adult stem cells such as mucous neck cell (half-life: 9–16 days) (Goldenring, Nam et al, 2011; Ramsey, Doherty et al, 2007; Suzuki, Tsuyama et al, 1983) or pit cells (half-life: 3 days) (Matsuo, Kimura et al, 2017) are generally shorter.

At first glance, one might think that because stem cells can self-renew and are therefore immortal, they have a long time to accumulate mutations over all those DNA replication events (Fig. 1A, upper left). However, even in organs with dedicated, mitotically active stem cells, there are reasons why stem cells may not be the most common source of would be less likely to transform into tumor cells. For one, it has been shown that epithelial stem cells can divide and differentiate stochastically, such that stem cells randomly can have multiple fates with each division. They can generate two stem cells or two differentiated cells or one of each (Fig. 1A, upper, middle and right) (Snippert, van der Flier et al, 2010; Zheng, Betjes et al, 2023). If stem cells divide into two progeny, both of which differentiate and stop replicating, the spontaneous mutations and other chromosomal abnormalities that arise in the stem cell would not be replicated unless the differentiated cells later revert to a stem cell state. If genetic material flows unidirectionally from stem cells towards elimination from the tissue (with no plasticity of differentiated cells), it would be difficult for chromosomal abnormalities to accumulate because mutations that arise would be transferred to cells destined to be differentiated and shed rather than maintained in the stem cell niche (Mills and Sansom, 2015; Simons and Clevers, 2011; Zheng et al, 2023). The premise that stem cell chromatin is immortal has also lost support: in the “immortal strand” theory, there must always be asymmetric segregation of chromosomes during self-renewal such that older DNA strands are retained in progeny stem cells with newly synthesized strands segregating to differentiated cells. But detailed studies of segregation show it to be variable in vitro, and whether asymmetric segregation even occurs at all in vivo is debated (Kiel et al, 2007; Lansdorp, 2007). Last, adult stem cell populations themselves are not as homogeneous and rapidly replicating as once assumed. A variety of long-term, short-term, and multipotent HSCs contribute to blood production during most of adulthood, and even highly dormant populations have been observed in various organs (Jost, 1986; Mysorekar, Isaacson-Schmid et al, 2009; Sun, Ramos et al, 2014; Wilson, Laurenti et al, 2008). All of these findings contradict the dogmatic belief that a single self-renewing bona fide stem cell generates “all” other lineages in a tissue, is immortal, and is the sole source of cancer. Differentiated cells are far more abundant than constitutively active, adult stem cells, are long-lived, can undergo plasticity events converting them into stem-like cells, and they can also autoduplicate without changing their identity (Fig. 1A, lower panels). All of those features contribute to their being highly underappreciated sources of cancer.

#### Differentiated cells are prone to polyploidy and multinucleation

**Figure 2:**
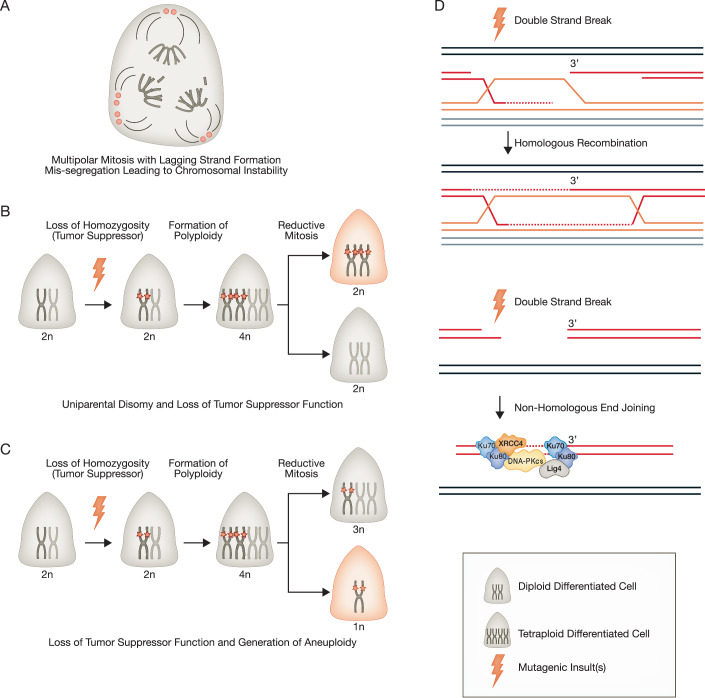
Factors resulting in differentiated cells with enhanced tumorigenicity. As differentiated cells are often polyploid or multinucleated, their mitosis may take several aberrant paths: (**A**) multipolar mitosis with lagging strand formation and mis-segregation leading to increased chromosomal instability; (**B**) uniparental disomy and premature loss of tumor suppressor function; (**C**) premature loss of tumor suppressor function and generation of aneuploidy. (**D**) Upon DNA damage, differentiated cells rely on error-prone DNA damage repair mechanisms such as nonhomologous end joining (lower) rather than error-free homologous recombination (upper), which is the only possible when sister chromosome is available.

Third, differentiated cells frequently become polyploid and multinucleated. Generation of polyploidy and multinucleation has been described across multiple species, especially those with long-lived differentiated cells, including hepatocytes, acinar cells in the pancreas and the salivary gland, trophoblast giant cells in the placenta, gastric chief cells, breast alveolar cells during lactation, and multiple cell types in the kidney cortex, such as podocytes and tubular epithelial cells (Fig. 1A, lower, center) (Chen, Ouseph et al, 2012; De Chiara, Conte et al, 2022; Fantone, Tossetta et al, 2022; Fox et al, 2020; Ge and Morgan, 1990; Kriesten, 1984; Lazzeri, Angelotti et al, 2018; Liapis, Romagnani et al, 2013; Matondo, Moreno et al, 2018; Nagata, Nakayama et al, 1998; Rios, Fu et al, 2016; Unhavaithaya and Orr-Weaver, 2012; Vidal, 1984; Wollny, Zhao et al, 2016; Zanet, Freije et al, 2010). On the contrary, multinucleation has, to our knowledge, not been reported in somatic stem cells (Donne, Saroul-Aïnama et al, 2020; Øvrebø and Edgar, 2018). In fact, multinucleation would contradict the canonical definition of a stem cell, which states that a stem cell must be capable of self-replication, which is a problem for polyploid cells that cannot easily replicate a progeny cell with the same polyploid state through normal mitosis. As we discussed above, even at homeostasis, differentiated cells can autoduplicate (Fig. 1A, lower, left). Disrupted division after duplication of DNA is the source of most polyploid or multinucleated cells, the generation of which is not necessarily abnormal and, indeed, may represent a kind of hyper-terminal differentiation such that polyploidy may be a functional adaptation for differentiated cells to enlarge and increase efficiency (Comai, 2005; Gorla, Malhi et al, 2001; Rancati, Pavelka et al, 2008; Rios et al, 2016; Unhavaithaya and Orr-Weaver, 2012). However, with repeated injury and inflammation, these hyperterminally differentiated cells can paradoxically increase the risk of developing cancer. One reason may be that multinucleated, differentiated cells in multiple organs across various species can divide, especially after injury (Fox, Gall et al, 2010; Frade, Nakagawa et al, 2019; Gjelsvik, Besen-McNally et al, 2019; Gladfelter, Hungerbuehler et al, 2006; Lucchetta and Ohlstein, 2017; Matsumoto, Wakefield et al, 2020; Wang, Yang et al, 2021). In mammals, the characteristics, mechanisms, and consequences of polyploidy and multinucleation have been arguably best studied in the liver. Hepatocytes exist not only as diploid (2n), but as polyploid: tetraploid (4n), octaploid (8n), or even 16n cells, that can divide via noncanonical, reductive mitoses (Guidotti, Brégerie et al, 2003; Matsumoto et al, 2020). With this kind of mitosis, an octaploid cell can give rise to two tetraploid cells, with other combinations also possible, such as two diploids and one tetraploid, or even four diploids (Duncan, Taylor et al, 2010; Matsumoto, Wakefield et al, 2021). To achieve such mitotic patterns, the number and positions of centrosomes must be different from those of conventional diploid mitosis, with potentially more segregation foci (Fig. 2A). If all such unusual segregation events are not properly coordinated, there is a high probability of lagging chromosomes and mis-segregation, resulting in daughter cells with chromosomal abnormalities (Cimini, Howell et al, 2001; Nicholson, Macedo et al, 2015). In addition, uniparental disomy, which is extremely rare in normal mitosis but can occur in differentiated cells, can predispose up to one-third of hepatocytes to loss of heterozygosity (Fig. 2B) (Duncan et al, 2010; Tuna, Knuutila et al, 2009). Finally, with a single division of a polyploid cell, progeny cells may lose heterozygosity of tumor suppressors (Fig. 2C). Indeed, it has been proposed that hepatocellular carcinoma can occur in polyploid hepatocytes through mechanisms including aberrant ploidy reduction of mature hepatocytes (Lin, Huang et al, 2021; Matsumoto et al, 2021; Potapova and Gorbsky, 2017). This polyploidy-related chromosomal aberration has been more obvious in the context of the absence of p53, as demonstrated in the *Trp53*^*−/−*^ mouse mammary epithelial cells (Fujiwara, Bandi et al, 2005). The chromosomal abnormalities that characterize cancer in adult tissues in general might often be the result of aberrant ploidy reduction in polyploid but otherwise normal phenotypically normal-appearing cells (Coward and Harding, 2014; Miyaoka, Ebato et al, 2012; Storchová, Breneman et al, 2006).

#### Differentiated cells use error-prone DNA damage repair mechanisms

Fourth, differentiated—versus constitutively dividing—stem cells are limited to more error-prone DNA damage repair mechanisms. While embryonic or adult stem cells can use homologous recombination (HR, Fig. 2D) to repair double-strand breaks (DSB) and thus ensure faithful repair (Choi, Yoon et al, 2020), long-lived, mitotically quiet differentiated cells use more error-prone methods such as nonhomologous end joining (NHEJ**)** or microhomology-mediated end joining (MMEJ), also known as alternative end joining (Alt-EJ, Fig. 2D) (Choi et al, 2020; Her and Bunting, 2018; Mujoo, Pandita et al, 2017). Cells can use HR only when dividing because a sister chromatid is needed as a template. Thus, HR occurs during S-phase and, to a lesser extent, G2 phase, and is available to actively dividing, constitutive stem cells (Johnson and Jasin, 2001; Mohrin, Bourke et al, 2010; Saleh-Gohari and Helleday, 2004). On the other hand, cells in G0 or G1, which are the cell cycle phases where differentiated cells reside almost exclusively, do not have sister chromatid templates and are more likely to use mechanisms like NHEJ (Fowler, Chen et al, 2022; Heyer, Ehmsen et al, 2010; Scully, Panday et al, 2019). NHEJ tends to result in small deletions or insertions because broken ends cannot be faithfully reconstructed without the template. Similarly, MMEJ mechanism typically result in mutations due to the loss of DNA between the two microhomology domains and because DNA repair by this mechanism uses Polymerase Theta, which lacks 3’- > 5’ proofreading capacity (Arana, Seki et al, 2008; Sfeir and Symington, 2015; Wyatt, Feng et al, 2016). Nucleotide excision repair is another well-known mechanism for fixing aberrant DNA, which is actively used in stem cells; its activity is known to decrease as they differentiate, as noted in the hematopoietic or nervous systems, making differentiated cells more vulnerable to accumulating mutations (Hsu, Hanawalt et al, 2007; Nouspikel, 2007; Nouspikel and Hanawalt, 2000; Rocha, Lerner et al, 2013). Moreover, even when differentiated cells divide and can repair DNA damage using HR, if they are polyploid, the HR pathway may lead to errors, as non-sister chromatids can be used as a template for “homologous” repair, which itself can lead to loss of heterozygosity (Fugger and West, 2016). Another check adult stem cells have on propagating mutations and chromosomal abnormalities is their increased sensitivity to senescence and programmed cell death if repair is compromised (Blanpain, Mohrin et al, 2011; Hua, Thin et al, 2012; Mandal, Blanpain et al, 2011; Potten, Owen et al, 2002; Rocha et al, 2013; Sotiropoulou, Candi et al, 2010), which is similarly noted in embryonic stem cells (Schumacher, Pothof et al, 2021; Tichy, Pillai et al, 2010). However, one variable in the mutation-repair equation is the rate of mutation accumulation in adult stem vs differentiated cells. It is not clear if precise values for relative mutation rates along differentiation axis have been studied in vivo to date, and, of course, differing rates of mutation accumulation can play a role in how quickly cells can repair genomic damage.

Thus, adult stem cells represent a tiny portion of the cells in the body and are present in only a few organs where high turnover necessitates continuous replenishment. Only a subset of adult stem cells may satisfy the definition of a dogmatic true stem cell, and those are equipped with an error-minimizing, reliable DNA damage repair system and increased sensitivity to senescence or death if damage is not repaired, overall making them less likely to be the source of cancer. Long-lived differentiated cells, on the other hand, (1) are abundant (Barker, Bartfeld et al, 2010; Challen, Boles et al, 2009; Pang, Loo et al, 2023), (2) have more time to accumulate mutations because they are quiescent and long-lived, (3) are prone to polyploidy and multinucleation, and (4) use more error-prone repair mechanisms in the G0 and G1 stages of the cell cycle where they mostly reside, making them more likely to accumulate mutational burden if they are eventually called back into the cell cycle.

Box 2 A glossary of the terms for cell fate determination and cellular plasticity

**Table:** 

Term	Definition
Plasticity	An innate potential to phenotypically change cell type or cell identity. Most frequently used to describe a process where a differentiated cell obtains the characteristics of another differentiated cell, a stem cell, or becomes a less-differentiated cell with new attributes (e.g., proliferation).
Metaplasia	A change in a given differentiated cell type to another cell type that does not normally exist in the organ. An example of cell plasticity. Usually defined by histopathological, not molecular, features of cells in a tissue
Autoduplication	Also known as self-replication, mitotic division of a differentiated cell ultimately giving rise to two differentiated cells of identical morphology and function. Does not require change in differentiation or return to a more progenitor-like state.
Differentiation	A term used to describe the process by which a cell acquires a robust organellar composition necessary to execute a specialized function in mature tissue; a unique phenotype noted in the multicellular organism where division of cellular labor occurs.
Dedifferentiation	A differentiated cell decreasing its differentiated characteristics functionally or morphologically to become more like the progenitor or stem cell from which the cell previously derived.
Transdifferentiation	The term is used to describe the cellular process by which one differentiated cell changes its identity to another differentiated cell, with or without going through the stage of the progenitor or stem cell-like phase.
Quiescence	A state of minimal proliferation, characteristic of differentiated cells at homeostasis but also a feature of some relatively undifferentiated cells that can be recruited for tissue repair after injury (e.g., satellite cells in skeletal muscle).
Senescence	State when cells decrease normal physiological functions and cease division without dying. Senescent cells also exhibit a stereotypical cohort of other features, including increased pro-inflammatory signaling.

### Examples of cell plasticity of differentiated cells in various organs

As discussed above, differentiated cells can maintain tissue homeostasis by simple autoduplication in which a mature, differentiated cell simply divides into two differentiated cells of the same type, possibly through dedifferentiation or subcellular organelle modification to a certain extent. (Brown, Cho et al, 2022; Puhka, Vihinen et al, 2007; Tata and Rajagopal, 2017). However, differentiated cells that proliferate, especially after tissue injury and inflammation, can also alter their identity to a more progenitor-like phenotype (Fig. 1B). By definition, such identity changing is a manifestation of cell plasticity. Plasticity can involve true dedifferentiation, in which the mature cells revert to a less-differentiated, progenitor state in which they can self-replicate as progenitors and then subsequently either redifferentiate into either the original differentiated parent state or redifferentiate into a different cell type (Mills, Stanger et al, 2019). Differentiated cells can also exhibit transdifferentiation, with the cell phenotypically changing from one differentiated cell lineage into another without a stem cell-like intermediate. Transdifferentiation can be used by a tissue to replace one cell type whose census has been depleted with another lineage that switches identity to replace the lost cells without necessarily any cell division. In the pancreas, for example, alpha cells in the islet can transdifferentiate to beta cells to replenish beta cells if they are ablated by means that spare alpha cells (Thorel, Népote et al, 2010). Likewise, although we discuss here in several contexts how injury triggers a boost in the plasticity of pancreatic acinar cells, which can lead them to reprogram into so-called acinar-ductal metaplasia (ADM) cells that are progenitor-like and proliferate, they can also give rise, in a potential transdifferentiation event, to non-dividing chemosensory tuft cells (DelGiorno, Naeem et al, 2020). In a mouse model with congenitally absent peripheral bile ducts, hepatocytes can transdifferentiate into cholangiocytes in the liver. (Schaub, Huppert et al, 2018). Conversely, functional hepatocytes can be generated from biliary cells in the model when hepatocyte proliferation is blocked concurrently with liver injury, or after severe hepatocyte ablation (Oderberg and Goessling, 2023; Raven, Lu et al, 2017). Hematopoietic cells can also transdifferentiate: for example, forced expression of the transcription factor C/EBPα turns B cells into macrophages (Bussmann, Schubert et al, 2009).

Numerous quiescent cell lineages also dedifferentiate and proliferate to regenerate the lung after injury, evidenced by terminally differentiated luminal secretory cells dedifferentiating to act as recruited “basal” stem cells (Kotton and Morrisey, 2014; Tata, Mou et al, 2013). Adult renal epithelial differentiated cells also divide after injury, likely via dedifferentiation, though the relative role of a strictly autoduplication of fully differentiated cells is unclear (Fujigaki, 2012). Transiently dedifferentiated tubular cells in the proximal tubule of the kidney have been shown to be involved in regeneration (Smeets, Boor et al, 2013). In the intestine, fully differentiated, quiescent Paneth cells, after irradiation, can gain multipotency and give rise to other types of cells (Yu, Tong et al, 2018). Mammalian salivary glandular cells can use autoduplication during homeostasis but are also capable of transforming into ductal cells or vice versa in conditions of extreme injury, such as irradiation (Weng, Aure et al, 2018). As a final example of a large literature on cell plasticity, dedifferentiation of follicular thyroid cells can lead to the emergence of multilineage progenitor cells (Suzuki, Mitsutake et al, 2011).

### Why is it important to recognize plasticity and the role of differentiated cells in generating tumors? (the trouble with the “Cancer Stem Cell” model)

Given the overwhelming abundance of differentiated cells, their proneness to mutation accumulation and long lives, and their propensity for plasticity and proliferation, a picture begins to emerge about how differentiated cells and plasticity must be studied to better understand adult tumorigenesis. In particular, as injury and inflammation greatly increase the plasticity of differentiated cells, allowing them to be recruited as progenitors to replace damaged tissue, we begin to understand how chronic inflammation and recurrent bouts of injury over the course of an organism’s lifetime would predispose it to develop cancer. Recurrent injury and dedifferentiation events provide a means for banking mutations in long-lived, DNA repair-incompetent, differentiated cells, increasing the likelihood of multiple chromosomal defects and mutations that can ultimately lead to cancer (Fig. 1C). With long intervals in G0, followed by expansion periods where differentiated cells are recruited back into the cell cycle to regenerate lost cells, they can accumulate mutations and slowly expand them within the tissue as the organism ages. As long as the cells can redifferentiate, despite the burden of mutations, there may be no effect on the physiological function of the differentiated cells. However, if a mutation or other genomic or epigenomic abnormality occurs that blocks the return to a quiescent, differentiated state, then uncontrolled expansion of aberrant clones can occur as the first chronic precancerous event. We have called this potential sequence of events in tumorigenesis the Cyclical Hit Model (Burclaff and Mills, 2018; Huang, Pang et al, 2023; Jin and Mills, 2019; Miao, Sun et al, 2021; Saenz and Mills, 2018).

The Cyclical Hit model can also explain how somatic mutational burden increases with age, even if tissue looks histologically normal. It has been shown that differentiated cells have more phenotypic constraints on their behavior and morphology than on the fidelity of their genotype, a concept termed canalization by Waddington (Waddington, 1942). Hence, as long as cells can return to a differentiated state after a regenerative phase, they may look histologically normal. An important example of that phenomenon of histology not correlating with genomic alteration load is manifested by the often surprisingly similar mutational burden of cells in malignant and surrounding chronically injured nonmalignant tissue (Shimizu, Marusawa et al, 2014; Weaver, Ross-Innes et al, 2014).

The oncogenicity of a specific genetic mutation is context-specific and varies dramatically by the cell type, which is a testament to the importance of extragenomic factors like chromatin state, methylation, and the presence of absence of intercalating signaling networks. Beyond genetic and non-genetic factors influencing the oncogenic penetrance of a specific mutation, the expression of a phenotype is also situational (Hodis, Triglia et al, 2022). The Cyclical Hit Model potentially explains one aspect of why specific genomic abnormalities are also situational. For example, long-lived, differentiated pancreatic acinar cells have been shown to eventually, through dedifferentiation and mutational events, become pancreatic ductal adenocarcinoma cells (Storz and Crawford, 2020). The first step on the route to cancer is often thought to be when recurrent tissue damage induces acinar cells to return to the cell cycle as progenitors via the ADM mentioned above, with ADM eventually not redifferentiating to acinar cells and leading to the emergence of proliferating clones that can progress to the histologically abnormal lesion Pancreatic Intraepithelial Neoplasia (PanIN) and thereafter to adenocarcinoma (Kopp, von Figura et al, 2012; Zhu, Shi et al, 2007). The majority of pancreatic ductal adenocarcinomas express mutant (constitutively active) KRAS that drives proliferation (Kanda, Matthaei et al, 2012). However, mouse models have demonstrated that mutant KRAS alone is not a significant driver of proliferation in acinar cells at homeostasis; however, it acts as a stronger driver of proliferation once they have been reprogrammed to ADM following an injury or other stimulus as compared to the homeostatic condition (Hingorani et al, 2003) Similar findings are noted during melanoma formation; expression of the BRAF^V600E^ mutation in zebrafish or iPSC-derived melanocytes is an inefficient route to tumorigenesis. However, expression of ATAD2 in melanocytes to switch the melanocytes to a cell more progenitor-like state (i.e., changing cell differentiation context) leads to increased melanoma formation (Baggiolini, Callahan et al, 2021).

Cellular differentiation context is also critical in the case of the most important genetic abnormality in adult solid cancer: dysregulation of the canonical tumor suppressor p53. Whereas alterations in p53 function had previously been thought to be a later step during tumorigenesis, it is becoming clear that p53 aberrations are often a critical early step, occurring in otherwise phenotypically normal cells. For example, in esophageal adenocarcinoma, loss of p53 function occurs in metaplastic cells that look no different from metaplastic cells with wild-type p53 function. However, such cells may have substantial genomic alterations (Nowicki-Osuch, Zhuang et al, 2021; Redston, Noffsinger et al, 2022; Stachler, Camarda et al, 2018; Weaver et al, 2014). Dysplastic cells with loss of p53 can progress to neoplasia with an almost choreographed sequence of further genomic abnormalities, suggesting that in each new cellular differentiation context en route to malignancy, p53 plays a different role (Baslan, Morris et al, 2022). Similar p53 contextual behavior has been shown in the progression of metaplastic cells in the stomach to gastric cancer (Sethi, Kikuchi et al, 2020). In mouse models of pancreatic ductal adenocarcinoma tumorigenesis, mutant p53 is used as a driver that induces metaplastic reprogramming of acinar cells such that mutant KRAS can subsequently cause expansion of the mutant cells. In the zebrafish melanoma model mentioned above, loss of p53 is critical for the dedifferentiation of melanocytes to neural crest progenitors that allows mutant BRAF to drive melanoma formation (Kaufman, Mosimann et al, 2016).

Accepting a substantial role for differentiated cell plasticity in tumorigenesis raises questions about how cancers behave. For example, if adult solid cancers arise from normal differentiated cell ancestors, they may generally behave more like differentiated cells than stem cells. Thus, perhaps we should reconsider the concept of so-called “cancer stem cells” that re-establish cancers, even after the bulk of tumor cells are destroyed. Rather, it may be possible that—just as in non-neoplastic tissue—the bulk of cancer cells possess differentiated features-like, but most such cells are capable of entering the cell cycle after injury. This perspective on cancer cell behavior is supported by the fact that tumors exhibit varying degrees of differentiation status (Madison, Liu et al, 2013; Suzuki, Masuike et al, 2021). Tumors may grow constitutively in part because: (a) their “differentiated” cells have either lower injury thresholds to induce return to the cell cycle (loss of p53 lowers that threshold in non-neoplastic cells, as noted above); and/or (b) tumor cells constitutively consider their environment to be inflammatory; (c) and/or the tumors themselves create and maintain basal high-inflammatory microenvironments. Specifically, cancer cells may cycle between the phenotype of a large, mitotically quiescent, differentiated-like cell with imprecise DNA repair mechanisms and that of an induced, regenerative state, where they proliferate. The mitotically quiescent, differentiated-like state would be protective from traditional chemotherapeutic agents that target dividing cells, as recently reported by Rehman (Rehman, Haynes et al, 2021). Finally, if tumors behave more like differentiated cells being recruited back towards a progenitor state, they may also often use more error-prone DNA repair mechanisms rather than HR, further compounding genetic abnormalities.

Another important issue with the cancer stem cell concept in view of a better understanding of plasticity in the formation and propagation of cancers: As most tissues do not have a professional stem cell in the first place, how would cancer of that tissue create a new stem cell-like cell specifically within a tumor? Unfortunately, if it is rather the case that tumors are composed of plastic differentiated-like cells—just as in normal tissue—there would seem to be little hope for prospectively isolating a specific population of tumor-seeding (cancer stem) cells, because nearly all the tumor cells, whether mitotic or not, will be capable of easily reprogramming after therapy to a proliferative state (Vendramin, Litchfield et al, 2021).

### A liability of being multicellular: the requirement for plasticity

Why did this error-prone, potentially tumorigenic process of plasticity to recruit regenerating cells after injury evolve? Why don’t all tissues feature genome-error-resistant, professional stem cells? Tumors are diseases of multicellular organisms, and multicellular organisms usually regenerate lost cells after injury so it is worth examining how multicellularity, regeneration, and tumors arose during evolution to see if insights can be gained about how plasticity and tumorigenesis are related.

**Figure 3:**
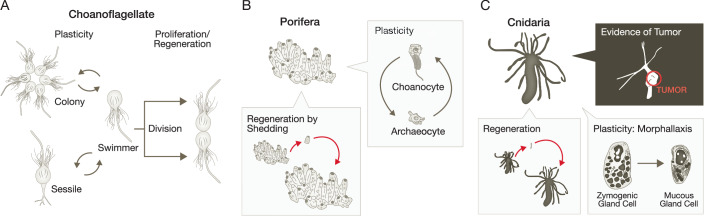
Evolutionary conservation of cell plasticity. (**A**) Choanoflagellates are unicellular organisms and the closest living relatives of metazoans. These single-celled organisms have a variety of differentiated organelles and the ability to divide (right). Choanoflagellates also exhibit temporal and spatial plasticity in their behavior throughout their lifetimes (left). (**B**) Porifera (sea sponges) are basal metazoans with a remarkable capacity for regeneration (left, note regeneration of whole organism from single shed cell) and highly plastic cell components (right). (**C**) Cnidaria, another basal metazoan, retain the ability to regenerate and become plastic, although multicellularity may be becoming more obligatory in this species, as it exhibits the earliest evidence of tumor formation and a functional p53 gene.

As detailed in Box 3, studying how organisms evolved to be composed of multiple cells can shed light on which cells are the ones originating cancer. Even choanoflagellates, the complex, unicellular organisms that are the closest relatives of the ancestor of all of us multicellular animals, are helpful in that they remind us that the original cell division process was not stem cell-based. Whereas constitutive, tissue stem cells in more complex multicellular organisms like mammals, are maintained and divide with mostly uncomplicated, unspecialized cytoplasms. They develop and expand specific organelles as they choose a cell lineage and differentiate. On the other hand, choanoflagellates must divide while maintaining elaborate features like flagella and extensive ER. Thus, the original eukaryotic cell division process resembles autoduplication of differentiated cells, indicating that maintaining a resident pool of undifferentiated, dividing stem cells is a more recent development in evolution. However, choanoflagellates also can dramatically vary their morphology and function under different conditions (Fig. 3A), going from multicellular, colony organizations to free swimmers similar (and perhaps ancestral to) how obligate multicellular organisms can have cells undergo programmed transitions from epithelial to migratory mesenchymal (epithelial to mesenchymal transition) during developmental stages like gastrulation (Newman, 2016). Such temporal-to-spatial transitions and division of labor drivers contribute cooperatively to maintaining cell specialization while allowing for cell plasticity in multicellular organisms. In this regard, the plasticity of multicellular organisms may simply be a more refined and enhanced reflection of structural and functional plasticity of precursor forms already evolved in complex unicellular organisms like choanoflagellates, that is also noted as a behavior of cancer cells during invasion and metastasis (Mittal, 2018; Yilmaz and Christofori, 2009).

Among the least complex multicellular organisms are the Porifera or sea sponges. They are composed of a limited number of cell lineages that show remarkable plasticity, along with the capability of even a single cell to reprogram to give rise to a whole, new multicellular organism (Fig. 3B, lower left). The more complex Cnidaria include hydra, which show more cell specialization and even hierarchical stem cell relationships with less-differentiated stem cells constitutively dividing and differentiating to give rise to different lineages (Bode, 2003). Even though hydra may have dedicated adult stem cells, they are also known for their remarkable plasticity, with a handful of cells able to regenerate whole organisms. Interestingly, regeneration can also occur by a process called morphallaxis, a sequential dedifferentiation and redifferentiation process, which does not require cell division (Bode, 2003; Bosch, 2007). The transdifferentiation of zymogen cells into granular mucous cells is a striking example of cell plasticity occurring through morphallaxis in this species (Fig. 3C, lower right) (Siebert, Anton-Erxleben et al, 2008). This reprogramming is remarkable because it is similar to a pattern of zymogenic to mucinous cell conversions seen in mammals in multiple organs: e.g., spasmolytic polypeptide-expressing metaplasia (SPEM) in gastric chief cells, the aforementioned ADM of pancreatic acinar cells, and reprogramming of acinar cells in salivary glands. The presence of zymogenic cells and adult stem cells with differentiated zymogenic cells maintaining plasticity suggests that even the specific manifestations of functional cell identity reprogramming of differentiated secretory cells in mammals have ancient origins in much simpler multicellular animals (Arendt, Musser et al, 2016; Haynes and Davis, 1969).

Taken together, it is clear that cell differentiation and the ability of those differentiated cells to autoduplicate as well as to drastically change identity, function, and proliferative potential are all features that evolved at the origin of multicellularity, if not sooner. Many organisms have the ability to regenerate appendages and even whole organisms from a handful of differentiated cells. Thus, the ability of differentiated cells to become proliferative and migrate to new niches where they then rapidly expand has been encoded in evolution, making it likely that cancers often co-opt this ancestral plasticity program.

#### Multicellularity, cancer, and the role of p53: an evolutionary perspective

Cancer is a disease of multicellular organisms (Aktipis, Boddy et al, 2015; Domazet-Lošo and Tautz, 2010). Malignant tumors are a disease because they draw resources from other cells, invade tissue, disrupt function of organs, and ultimately lead to abnormal function or death of the organism as a whole. Thus, multicellularity is a pre-requisite for tumorigenesis, but not all multicellular organisms are prone to tumors. In fact, for example, there is no documented evidence of malignancy in the sea sponge (Porifera species), despite the fact that they can live hundreds or even thousands of years (Jochum, Wang et al, 2012; Risk, Heikoop et al, 2002). Rather, such organisms, like the desmosponge *Tethya Wilhelmina*, can tolerate up to 600 Gy of radiation, demonstrating that sponges may be extremely resistant to radiation (Fortunato, Taylor et al, 2021b). Such basal metazoans are closer to facultative multicellular organisms, and they may avoid cancer because they simply shed off single cells that contain mutations and regenerate the entire organ from the remaining healthy cells (Fig. 3B). In fact, shedding is one of the well-known characteristics of Porifera, as observed in the marine sponge *Halisarca caerulea*, which is capable of increasing proliferation to generate and shed choanocytes (De Goeij, De Kluijver et al, 2009). Ctenophora and Placozoa also have not been documented to form tumors. Intriguingly, like Porifera, Placozoa *T. adherens* displays extraordinary radiation resistance (tolerance up to 218.6 Gy) and the ability to shed inviable cells (Fortunato, Fleming et al, 2021a), indicating these organisms are still not entirely committed to multicellularity and thus not highly susceptible to cancer.

Cnidarians, on the other hand, have been shown to develop cancer (Fig. 3C, upper) (Domazet-Lošo, Klimovich et al, 2014). The female gametes of *Hydra oligactis* and *Pelmatohydra robusta* can become tumors that can be transplanted and reduce the organism’s fitness (Fig. 3C, upper). Although the phylogenetic determination of being more basal to another species appears to require more consideration (Collins, Cartwright et al, 2005), the increasing diversity of specialized cells and tissue (e.g., germ layers) in Cnidarians, along with the first evidence of cancer in this species, may be consistent with the idea that cancer becomes a greater problem as evolution advances toward more obligatory multicellularity. Accordingly, phylostratigraphic analysis demonstrates that the evolution of proteins that can act as tumor suppressors coincides with the onset of multicellularity, which has significant implications for the development of tumors (Domazet-Lošo and Tautz, 2010; Trigos, Pearson et al, 2019).

To better understand the relationship between evolution of multicellularity and evolution of genes that regulate tumorigenesis, we explore the evolution of arguably the most important tumor suppressor, p53. Proteins with significant sequence homology to the p53 family (comprising p53, p63 and p73) evolved as early as the more complex unicellular organisms like the Choanoflagellate, *Monosiga brevicollis* (Nedelcu and Tan, 2007), or the Filasterea, *Capsaspora owczarzaki* (Sebé-Pedrós, de Mendoza et al, 2011), suggesting their conservation to the last common ancestor of holozoans. However, no evidence of p53-like functionality in these species has been found; in other words, these proteins have not been documented to regulate death or senescence or response to DNA damage. Evidence of p53 orthologs exists only sparsely among basal, multicellular metazoans; they are thought to be absent in Porifera (Åberg, Saccoccia et al, 2017), while both p53 and MDM2 genes are found in the Placozoa, *Trichoplax adhaerens* (Lane, Cheok et al, 2010). It is intriguing that only the MDM2 transcript seems to increase in response to DNA damage, and not p53 in Placozoa (Fortunato et al, 2021a). This sparsity and functional dissimilarity of p53 and related factors in basal metazoans and unicellular organisms may indicate that the importance of p53 matches the evolution of tumor susceptibility, both of which are of higher importance in organisms that are obligatorily multicellular.

Overall, studying evolution with an eye toward plasticity, tumorigenesis and multicellularity, a pattern emerges. Organisms that develop tumors and, thus, need tumor suppressors, are those with complex tissue patterns in which there is plasticity but in which regeneration via plasticity is more restricted, such that the organism cannot be regenerated by a single, shed cell. The problem with cells in multicellular organisms having the ability to return to the cell cycle is that some cells may accumulate excessive mutations or may be unable to return to a differentiated state after repetitive injury. Such quasi-differentiated, proliferative cells, if not shed, can form tumors and jeopardize the health of the whole organism. It has been proposed, in fact, that organs that generate and shed cells constantly may also be protected against cancer (Hammarlund, Amend et al, 2020). Licensing differentiated cells to obtain these potentially dangerous cell states is a fundamental problem with multicellularity and regulation of these states is critical for the survival of the organism (Aktipis et al, 2015; Aktipis and Nesse, 2013; Davies and Lineweaver, 2011; Zhu, Wang et al, 2022). It is not clear if there are any multicellular organisms that are cancer-susceptible but that have only differentiated cells. If so, those would theoretically be the best organisms to study a purely differentiated cell origin of cancer. In any case, it is important to point out that both stem cells and differentiated cells can serve as a source of cancer, even if we focus in the current manuscript on reasons why differentiated cells have been understudied in this regard.

Not surprisingly, we observe the first evidence of functional p53 orthologs: nvp63, nvpEC53, and nvpVS53 in *Nematostella vectensis* (Pankow and Bamberger, 2007), a Cnidarian, the species in which evidence of tumor formation is first observed (Domazet-Lošo et al, 2014). Nvp63, which has the highest gene structural similarity to the vertebrate p53 gene family, has all three major protein domains (transactivation domain, DNA binding domain, and oligomerization domain) of human p53 family member, p63. Nvp63 also binds to the vertebrate consensus *cis*-regulatory elements in mammalian p53 target genes and transactivates target gene transcription in vitro (Pankow and Bamberger, 2007; Rutkowski, Hofmann et al, 2010). Further, nvp63 is functionally similar to human p53 in that it is critically involved in UV-induced apoptosis of the early gametes, suggesting that p53 injury response evolved even in the earliest metazoans (Pankow and Bamberger, 2007) and that natural selection in these organisms favored a genetic mechanism for self-deletion of damaged cells.

Throughout evolution, we notice diversification with gene duplication events in the p53 superfamily; however, with general evolutionary conservation of its structure and function (Joerger and Fersht, 2016; Rutkowski et al, 2010). In *Drosophila Melanogaster* and *Caenorhabditis elegans*, a p63/p73 hybrid gene exists (respectively, Dmp53 and CEP-1) that is phylogenetically closer to nvpEC53 in *N. vectensis* (Pankow and Bamberger, 2007). The role of Dmp53 is also comparable to that of human p53 in that it participates in apoptosis induced by genotoxic stress (e.g., X-ray irradiation), although cell cycle progression is not affected (Ollmann, Young et al, 2000). CEP-1 is involved in the hypoxia-induced death of *C. elegans* germ cells (Derry, Putzke et al, 2001), which is the only cell type that retains proliferative potential in adults of this species (Albert Hubbard, 2007; Pintard and Bowerman, 2019), and may be the only organ that can be used to assess the function of p53.

To date, the cellular differentiation context of p53 (i.e., whether in active stem cells, mitotically quiescent differentiated cells or in differentiated cells recruited by injury into the cell cycle) has seldom been described (Almog and Rotter, 1997; Belyi, Ak et al, 2010; Solozobova and Blattner, 2011). However, evolutionary evidence described in this review clearly indicate that, throughout metazoan evolution, the p53 family emerges in ‘obligatory multicellular’ organisms whose differentiated cells can be induced to proliferate after injury, and p53 seems to play little or no role in organismal development or homeostatic tissue maintenance. In other words, p53 may not be required for developmental or tissue-maintaining, professional stem cells or differentiated cells in an uninjured state. Rather, the conserved function of p53 seems to be to police cell health after an injury that might call differentiated cells back into the cell cycle (Amit, Takahashi et al, 2020; Miao, Lewis et al, 2020). For all the reasons outlined above, differentiated cells are at high risk for genomic compromise and, thus, need a policing mechanism, like p53-regulated pathways; otherwise, the cell plasticity inherent to many differentiated cells puts them at great risk for spawning dysregulated growth.

Given that harnessing differentiated cells as a vector for regeneration seems to be a primordial and nearly universal feature of multicellular organisms, it is unsurprising that constitutively present, multipotent, undifferentiated cells seem to be rare features of tissues and organisms. Maintaining cells in a non-functional state only for the purposes of continuously making and shedding short-lived progeny is energetically expensive when compared to devoting all cells to differentiated, functional activity and calling on those cells to regenerate damaged tissue only after injury actually occurs. From this perspective, professional, dedicated adult tissue stem cells are a luxury for multicellular eukaryotes, likely evolving only once much more complex organisms emerged in which a broad spectrum of differentiated cell types work together with some being highly specialized.

### Paligenosis: an evolutionarily conserved program used by differentiated cells to return to a proliferative, stem-like state

**Figure 4:**
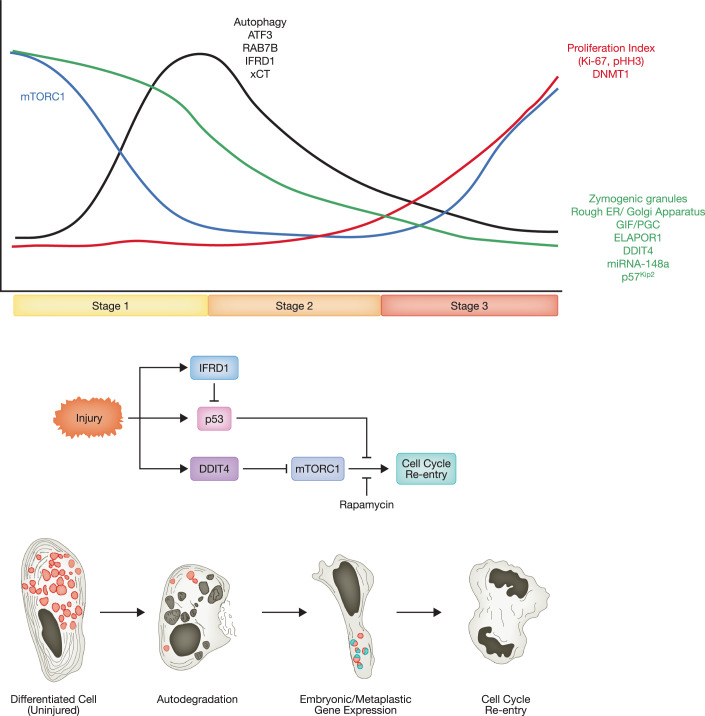
Paligenosis at a glance. Increased cell plasticity in mammalian cells after injury occurs through mechanisms that have existed throughout the evolution. Zymogenic cells of the gastrointestinal tract undergo a three-stage, stepwise process, which involves autophagy (stage 1), metaplastic/embryonic gene expression (stage 2), and proliferation (stage 3). Paligenosis is orchestrated by interplay among various factors, including mTORC1, p53, IFRD1, and DDIT4. mTORC1 mechanistic target of rapamycin complex 1, IFRD1 Interferon-related developmental regulator 1, DDIT4 DNA damage-inducible transcript 4.

Given the widespread pattern of plastic differentiated cells with evolutionary roots to early metazoans like Cnidaria, evidence has been emerging for evolutionarily conserved cellular programs whereby mature mitotically quiescent cells can re-enter the cell cycle. Though there are likely to be several such programs, similar to how there are multiple forms of programmed cell death (apoptosis, ferroptosis, etc.), to our knowledge, only one cellular plasticity program has been completely outlined. This stepwise molecular-cellular process that functions to scale down differentiated cell architecture for eventual return to the cell cycle has been termed “paligenosis” (from Greek *pali/n/m* = “return to” + *genes* = “generative” + “*osis*” = suffix for a cellular process) (Fig. 4) (Brown et al, 2022; Willet, Lewis et al, 2018).

Based on studies comparing multiple cell types in different organs to extract common features of this plasticity program, paligenosis begins with remodeling of existing cellular architecture in a process that involves massive upregulation of autophagolysosomes (Stage 1) (Willet et al, 2018). Genetic or pharmacological inhibition of lysosomal hydrolases blocks the generation of a proliferative, dedifferentiated cell, so this initial stage is required for paligenosis (Radyk, Spatz et al, 2021). As this autodegradative phase subsides, cells begin to express markers of embryonic/metaplastic cells (Stage 2), such as CD44 and certain SOX proteins (often SOX9). Finally, in Stage 3, cells re-enter the cell cycle. Just as lysosomes are needed for Stage 1, mTORC1 activity is required for cells to progress from Stage 2 to 3. Given the evolutionary pressures (discussed above) balancing regeneration and cancer and the risk of copying and thus perpetuating mutated DNA, it is not surprising that the transition to Stage 2 and to Stage 3 are gated by checkpoints which prevent unrestricted licensing to proliferate without first checking for competency and genetic stability (Miao et al, 2020; Miao et al, 2021).

Adami predicted more than a century ago that injury would coax some differentiated cells to reprogram into dividing cells and that the key feature of this reprogramming would be to cease the cell’s use of its energy to do adult, physiological functions and then to rewire cellular energetics to promote division. In accord with this hypothesis, in large, differentiated secretory cells, mTORC1 is active in routing energy into translating secretory cargo. In Stage 1, mTORC1 is shut off to stop secretion and permit autophagic dismantling of the differentiated cell. In Stage 2 to 3 transition, it is required to ramp up again for cells to enter S-phase of mitosis. Much of the early (Stage 1) suppression of mTORC1 occurs via the scaffolding protein DDIT4 (aka REDD1). Normally, p53 continues suppression of mTORC1 as DDIT4 is degraded during Stage 2. Thus, it is the evolutionarily conserved tumor suppressor p53 that plays the critical fidelity check role. In the absence of DDIT4, paligenosis proceeds through Stage 1 and 2 relatively normally, but cells ignore the fidelity check before entering the cell cycle, such that *Ddit4*^*−/−*^ cells enter the cell cycle despite unrepaired DNA (Miao et al, 2021). That likely happens because p53 works by maintaining suppression of mTORC1 started by DDIT4. Without DDIT4, mTORC1 is never suppressed in the first place, and the error check stage is avoided. As might be predicted, *Trp53*^*−/−*^ cells have similar phenotypes to those of *Ddit4*^*−/−*^ cells (Miao et al, 2020).

Another key paligenosis-regulating protein is ATF3, which is induced rapidly after paligenosis-causing injury and plays a key role in inducing the autodegradative machinery, including the lysosomal trafficking protein RAB7 (Radyk et al, 2021). Its role seems predominately to manage Reactive Oxygen Species (ROS) generated in the initial, paligenosis-inducing injury as cells lacking the ability to manage ROS undergo ferroptotic death by Stage 3 (Miao et al, 2024). IFRD1 is upregulated during Stage 1 and 2 but is critical for eventual suppression of p53 and thus, in turn, derepressing mTORC1 to allow entry into S-phase. The function of IFRD1 is conserved even when *D. Melanogaster* enterocytes in the gut are injured and recruited back into the cell cycle (Miao et al, 2020) (Park and Mills, unpublished observations) as well as in Porifera (*T. wilhelma)* after radiation exposure (Fortunato et al, 2021b). Even the plant IFRD1 ortholog is involved in adaptation to injury (Park, Chung et al, 2009a). A series of other genes have also been elucidated as playing roles in various stages of paligenosis, though most have been studied in only one cell type and so may not be generally applicable to paligenosis. For example, it has been shown that cells in Stage 1 must respond successfully to challenge by oxidative species, and reactive oxygen species may be an initial triggering event (Meyer, Engevik et al, 2019). Stage 1 also involves decreasing genes that maintain the differentiated state like p57 and, in the case of secretory cells, MIST1 (aka BHLHA15) (Lee, Kim et al, 2022). Double-stranded RNA accumulates in Stage 1, which must be cleared by a mechanism involving the RNA-editing protein ADAR1 (Sáenz, Vargas et al, 2022).

From an evolutionary perspective, the pressures selecting for plasticity programs like paligenosis would be: (1) giving organisms the capacity to devote nearly all cells to differentiated function while still being able to call on those cells as progenitors after injury; (2) a requirement for licensing those cells to ensure they do not have genomic alterations that would cause them to expand indefinitely and threaten overall organism viability (see ref. Brown et al, 2022). It is unclear yet how widespread the full, 3-step program of paligenosis is among species and organs, because it has only recently been characterized. However, each of the stages of paligenosis have been broadly studied in plasticity and regeneration. For example, the requirement for autophagic and lysosomal activity has been shown to be required for differentiated cells to change phenotype in numerous settings (e.g., Song, Liu et al, 2020) (Johnson, Parham et al, 2022) and reviewed in Brown et al, 2022). Modulation of TOR activity has been shown to be required for numerous examples of cell plasticity. Specific genes like IFRD1 play roles in cellular injury response diversely from *Hydra* to *Arabidopsis* (Park, Chung et al, 2009b, Wenger, Buzgariu et al, 2014). A critical role for mTORC1 in regeneration has been noted across bilaterian metazoans, including platyhelminthes, teleosts, and amphibians (reviewed in (Ricci and Srivastava, 2018)) and even non-bilaterian Cnidarians (Brunkard, 2020; Chera, Ghila et al, 2011; González-Estévez, Felix et al, 2012; Lund-Ricard, Cormier et al, 2020; Peiris, Weckerle et al, 2012; Song et al, 2020). Many tissues across vertebrates require mTORC1 for regeneration, e.g.,: blastema formation and subsequent regeneration of fin (Hirose, Shiomi et al, 2014), and recruitment of mitotically quiescent mouse satellite cells to regenerate muscle (Rodgers, King et al, 2014). Recruitment of hepatocytes for regeneration after partial hepatectomy requires IFRD1 and mTORC1 (Fouraschen, de Ruiter et al, 2013; Miao et al, 2020).

Note that there are signaling pathways with critical roles in promoting paligenosis in various contexts (eg, Notch and Wnt), but these may be specific to certain tissues and species as signals to induce paligenosis, because they do not seem to be universal aspects of the cell-intrinsic paligenosis machinery.

Box 3 Evolution of multicellularity and evolution of cancer cells of origin Choanoflagellates are “unicellular” organisms that are the closest living relatives of metazoans, with widely studied genetic and phenotypic characteristics that provide unique and significant opportunities to investigate the origins of multicellularity (Fairclough, Chen et al, 2013; King, Westbrook et al, 2008; Laundon, Larson et al, 2019; Levin, Greaney et al, 2014). It is a well-supported supposition that the first animal-like, differentiated cell types were likely similar to choanoflagellate “collar cells” with an apical flagellum and multiple sophisticated organelles (Fig. 3A) (Brunet and King, 2017; Richter and King, 2013). Each choanoflagellate has distinct organelles such as the flagellum and endoplasmic reticulum, showing that urmetazoan ancestors were unicellular creatures with abundant differentiated organelles, in contrast to undifferentiated adult stem cells in mammals (Laundon et al, 2019). Further, this unicellular organism can become functionally multicellular through division and incomplete cytokinesis, with help of extracellular matrix synthesis through C-type lectins and cadherins (Abedin and King, 2008; King, Hittinger et al, 2003). Those cell-cell interaction and adhesion proteins, as exemplified by *Salpingoeca rosetta*, allow spontaneous formation of rosettes of single cells that are induced by cells sensing lipids from the choanoflagellate prey: bacterial species *Algoriphagus* (Fig. 3A) (Alegado, Brown et al, 2012; Levin et al, 2014).

Given choanoflagellates have complex, differentiated organelles, yet still must divide, these ancient, unicellular animal-like cells behave more like differentiated cells in a multicellular organism than they do like adult tissue stem cells. In other words, reversion to a less-differentiated, stem-like state with high nuclear:cytoplasmic ratios and relatively few specialized organelles is not a requirement for the division of this organism. Rather, ancestral, unicellular eukaryotic cell division resembles the autoduplication of differentiated cells. However, choanoflagellates do not remain rigidly locked in a single differentiated state, as they can drastically change morphology and functional focus over time, exhibiting a sort of temporal plasticity, often referred to as the temporal-to-spatial transition hypothesis (Brunet and King, 2017). Choanoflagellates, for example, can also form chain colonies as mitotically quiescent, multicellular-like cell clusters and can reprogram between various dramatically different states. Throughout their life cycle, they can be fast or slow swimmers, sedentary thecate cells glued to a surface and fully “differentiated” choanoflagellates can become amoeboid by retracting their flagella and activating myosin-based motility (Fig. 3, upper left) (Brunet and King, 2017; Dayel, Alegado et al, 2011).

Porifera, or sea sponges (Fig. 3B), are an ancient and primitive phylum of “multicellular” organisms (Srivastava, Simakov et al, 2010). Each organism has a limited number of differentiated cell types: archaeocytes, choanocytes, pinacocytes, myocytes, sclerocytes, and granular cells that resemble the secretory cells of mammals (Borisenko, Adamska et al, 2015; Müller, 2006; Schröder, Perović-Ottstadt et al, 2004). Archaeocytes mirror modern embryonic stem cells in that they are totipotent (Müller, 2006; Weissenfels, 1989), while pinacocytes, that line up the outer surface and internal canal of sponges, and choanocytes, which resemble choanoflagellates morphologically and work to absorb food, are similar to differentiated cells of mammals (Manconi and Pronzato, 2015). Recent transcriptomic analysis has shown that overall choanoflagellates are similar to the totipotent archaocytes despite their morphological resemblance to choanocytes (Diaz, 1977; Funayama, 2013; Gaino, Manconi et al, 1995; Hibberd, 1975; Sogabe, Hatleberg et al, 2019; Sogabe, Nakanishi et al, 2016); however, it was also demonstrated that choanocyte and pinacocytes can readily become archaeocytes and vice versa. It has been claimed that the plasticity of the differentiation states of cells dominates over a “point-of-no-return” differentiation in sponges; in other words, differentiated cells retain a remarkable degree of plasticity potential throughout sponge life (Müller, 2006). Porifera has also been known for its remarkable plasticity in terms of regenerative potential: dissociated Porifera cells in suspension are capable of aggregation and eventual regeneration of the complete organism as an extreme example (Eerkes‐Medrano, Feehan et al, 2015; Lavrov and Kosevich, 2014) (Fig. 3B, upper left), where increased plasticity should play a major role.

Cnidarians, another well-known basal metazoan phylum (Fig. 3C), also have remarkable plasticity and regeneration potential, which has been extensively studied in two species, *Nematostella vectensis* and *Hydra vulgaris*. As a multicellular organism, Hydra is composed of endoderm,ectoderm, and mesoglea (interstitium). Self-renewing, multipotent stem cells in the mesoglea produce differentiated cell types, including cnidocytes (nematocytes), secretory cells, neurons, and gametes, while a single layer of ectodermal and endodermal epithelial cells line the outer and inner (gut) lumen. These epithelial cells, while proliferating continuously, also execute physiological functions (protection or digestion) until they are shed, suggesting that a stem cell dedicating itself solely to division and differentiation (without physiological function) may be a later invention during evolution (Bode, 2003). Just as in Porifera species, cell plasticity potential is widespread in Hydra (Bosch, 2007). Indeed, since the 18th century, it has been recognized that Hydra exhibits amazing regenerative potential as clusters of as few as 5–15 cells from an organism can regenerate a whole body (Fig. 3C, upper left) (Gierer, Berking et al, 1972; Technau, Cramer von Laue et al, 2000; Trembley, 1744).

Placozoa and Ctenophora, two other basic multicellular metazoans, also possess the capacity for whole-body regeneration and plasticity. Although the existence of stem cells in Placozoa *Trichoplax adherens* is uncertain (Guidi, Eitel et al, 2011; Smith, Varoqueaux et al, 2014), the ability to regenerate the entire body suggests that the plasticity of differentiated cells may play a key role. Similarly, Ctenophora*Vallicula Multiformis* is able to regenerate its entire body from small peripheral tissues (Freeman, 1967). As continuously proliferating cells are observed locally only on the oral side (Jager, Dayraud et al, 2013; Schierwater, 2005), differentiated cell proliferation and plasticity are likely important for regeneration, which actively occurs (Tamm, 2012) in this phylum. Hence, evidence from evolution supports the notion that cellular plasticity has always existed and has been a routine mechanism exploited throughout evolution.

As multicellular organisms have grown more complex during evolution, regenerative potential has tended to decrease, though it has persisted to varying degrees in numerous bilaterian species; e.g.,: arm regeneration in echinodermata *Amphiura filiformis* (Czarkwiani, Ferrario et al, 2016), whole-body regeneration of the hemichordate *Saccoglossus kowalevskii*, (Tweedell, 1961) replacement of lost body parts through epimorphosis and morphallaxis in annelids *Lumbriculus variegatus* (Drewes and Fourtner, 1990; Martinez et al, 2005), limb regeneration in arthropoda *Parhyale hawaiensis*, (Konstantinides and Averof, 2014), whole-body regeneration in platyhelminths such as *Schmidtea mediterranea* (Benham-Pyle, Brewster et al, 2021; Montgomery and Coward, 1974) and structural regeneration in axolotl (Kragl, Knapp et al, 2009).

## Conclusions and perspectives

Here we have examined the intertwining of cellular plasticity programs and cancer and speculated about how these might have co-evolved. We also focused on the concept of paligenosis in which cells have access to a conserved plasticity program and potentially a suite of genes that execute and license it. Not surprisingly, the critical tumor suppressor, p53—which has evolved more or less as multicellularity, tumor susceptibility, and plasticity have evolved— also plays a central role in licensing cells during paligenosis. Thus, the evolutionarily conserved aspects of paligenosis appear both to be the mechanisms that convert a differentiated, mitotically inactive cell to a more progenitor-like, dividing cells and those that license the recruitment of that cell back into the cell cycle.

That there might be a shared program for this to occur (e.g., paligenosis and other undiscovered mechanisms) has several implications. For one, in most adult tumors, there are precursor states characterized by alterations in the normal histology in the presence of chronic inflammation/injury. Often the changes are recognized as metaplasia. If these lesions form by paligenosis of mature cells, then there might be a conserved set of genes that lead to precancerous lesions across multiple organs. Some evidence for this is emerging in that it has been shown that there are remarkable similarities among Barrett’s esophagus, intestinal metaplasia in the stomach, pancreatic acinar-to-ductal metaplasia, and serrated adenomas in the colon (Adkins-Threats and Mills, 2022; Chen, Scurrah et al, 2021; Goldenring and Mills, 2022; Ma, Lytle et al, 2022).

Another implication, touched on above, is that plasticity programs like paligenosis can offer new approaches into therapy against existing tumors. If tumors form from ancestor cells that failed paligenosis error-checking, tumors may thus persist by constantly re-invoking that aberrant program. Thus, rather than looking for “cancer stem cells”, we might target core paligenotic machinery to help lock cells in either the “differentiated” state (where they will not grow or invade) or in the cycling state (where they will be susceptible to anti-cell-cycle therapy). An advantage of targeting paligenosis, is that—as opposed to mitosis—paligenosis occurs in normal tissue only after injury, so, relative to most generalized anti-cancer therapeutic approaches that target all dividing cells, a paligenosis therapeutic approach might cause reduced side effects to normal stem cells.

Why has it taken so long to appreciate conserved cellular plasticity programs whereas programs to execute programmed death (eg apoptosis) have been known for decades? It is likely that we have missed paligenosis-like plasticity programs because most of our understanding of shared cell biological processes has been done on cells in tissue culture, most of which are cancer cell lines. To “grow” differentiated cells in culture is an oxymoron, because differentiated cells are mitotically quiescent and thus can’t be grown. Work on the cell biology of plasticity has been done in whole organisms. Whereas genetic and developmental biological questions are best answered in tissue, it is difficult to study stepwise, intracellular processes in vivo. Thus, in vitro systems (perhaps using organoids and specific differentiation/anti-growth culture conditions) are desperately needed for the field to advance.

Injury can induce plasticity. If error-checking fails in plasticity, it can lead to tumorigenesis. However, another outcome under the same circumstances is cellular senescence: rather than unsuppressed p53 leading to apoptosis, it can lead to the semi-permanent cellular senescent state. Senescent cells are injured differentiated cells that retain permanent, unrepaired DNA, even jettisoning γH2AX-associated chromatin fragments from the nucleus into the cytoplasm. It will be interesting to see if, as organisms age, they become less able to invoke paligenosis to repair tissue with cells tending to enter senescence instead. Teleologically, the pattern makes sense, because genomic damage increases as somatic cells age, (discussed above), so paligenosis should become riskier. Thus, cancer risk increases. On the other hand, if aging begins to result in an ever higher threshold for cells to undergo paligenosis vs senescence, the cancer risk can decrease. Unfortunately, the decreased regenerative potential that drives the decreased risk may also lead to organ failure with age as tissue can no longer use paligenosis to recruit stem cells.

Clearly, we are just at the beginning of incorporating cell plasticity into our understanding of some of the most important processes in human health. As we better understand the underlying mechanisms that govern plasticity, we can expect to make large leaps in our ability to target the outcomes we want to promote healing and prevent cancer and aging.
